# In vitro to clinical translational pharmacokinetic/pharmacodynamic modeling of doxorubicin (DOX) and dexrazoxane (DEX) interactions: Safety assessment and optimization

**DOI:** 10.1038/s41598-023-29964-4

**Published:** 2023-02-22

**Authors:** Hardik Mody, Tanaya R. Vaidya, Sihem Ait-Oudhia

**Affiliations:** 1 Center for Pharmacometrics and Systems Pharmacology, Department of Pharmaceutics, College of Pharmacy, University of Florida, Florida, USA; 2 Quantitative Pharmacology and Pharmacometrics (QP2), Merck & Co., Inc, Kenilworth, NJ USA

**Keywords:** Computational models, Cancer prevention

## Abstract

Despite high anticancer activity, doxorubicin (DOX)-induced cardiotoxicity (DIC) limits the extensive utility of DOX in a clinical setting. Amongst various strategies explored, dexrazoxane (DEX) remains the only cardioprotective agent to be approved for DIC. In addition, altering the dosing regimen of DOX has also proved to be somewhat beneficial in decreasing the risk of DIC. However, both approaches have limitations and further studies are required to better optimize them for maximal beneficial effects. In the present work, we quantitatively characterized DIC as well as the protective effects of DEX in an in vitro model of human cardiomyocytes, by means of experimental data and mathematical modeling and simulation (M&S) approaches. We developed a cellular-level, mathematical toxicodynamic (TD) model to capture the dynamic in vitro drug-drug interaction, and relevant parameters associated with DIC and DEX cardio-protection were estimated. Subsequently, we executed in vitro*-*in vivo translation by simulating clinical PK profiles for different dosing regimens of DOX alone and in combinations with DEX and using the simulated PK profiles to drive the cell-based TD models to evaluate the effects of long-term, clinical dosing regimens of these drugs on the relative cell viability of AC16 and to determine optimal drug combinations with minimal cellular toxicity. Here, we identified that the Q3W (once every three weeks) DOX regimen with 10:1 DEX:DOX dose ratio over three cycles (nine weeks) may offer maximal cardio-protection. Overall, the cell-based TD model can be effectively used to better design subsequent preclinical in vivo studies aimed for further optimizing safe and effective DOX and DEX combinations to mitigate DIC.

## Introduction

Doxorubicin (DOX), which belongs to the class of anthracyclines, is one of the most effective antineoplastic agents. It is widely prescribed, as a single agent or in combination with other chemotherapeutics, for a variety of solid tumors (breast, ovarian, thyroid, gastric, lymphomas, sarcomas, Wilm’s tumor, neuroblastoma, along with others); as well as for hematological malignancies (leukemias)^1–3^. However, the development of dose-limiting and acute or chronic cardiotoxicity is considered a serious complication with DOX therapy that severely limits its clinical utilization^4,5^. Depending upon the timing of its clinical manifestation, DOX-induced cardiotoxicity (DIC) can be classified as acute (occurs during or immediately after administration), early-onset (occurs within one year of administration) or late-onset (occurs after one year of administration) chronic cardiotoxicity. Previous studies have reported that a cumulative DOX dose of > 350–400 mg/m^2^ can cause DIC including a decline in the left ventricular ejection fraction (LVEF) while a cumulative dose beyond 550 mg/m^2^ can cause an increased risk of congestive heart failure^4,5^. While DIC is not completely understood, there are a plethora of studies that propose DIC to be a multi-step process with the involvement of multiple pathways that eventually lead to the death of cardiomyocytes^2,3,6,7^. These include mitochondrial oxidative stress, iron metabolism through complex formation, dysregulation of calcium homeostasis, lipid peroxidation, sarcomeric structure alterations, mitochondrial DNA damage, genetic modulations, apoptosis, along with others^8–14^.

Since various mechanisms have been proposed to be involved in the development of DIC, accordingly several strategies have also been investigated to counter the same^7^. These include the use of the iron chelator—Dexrazoxane (DEX, ICRF-187)^15,16^, angiotensin converting enzyme (ACE) inhibitors^17^, antioxidants including several vitamins (E and C)^18^, α-1 adrenergic receptors (AR) agonists, modification of DOX dosing regimens such as dose fractionation strategies as well as liposomal DOX formulation^19–21^. However, the clinical application for most of these strategies have been somewhat limited, and/or improvement in clinical cardiac safety, if any, has been suboptimal. Of them, DEX, a bisdioxopiperazine derivative, is the only drug clinically approved as a cardioprotective agent, to treat anthracycline-induced cardiomyopathy as well as doxorubicin-induced extravasation^22,23^. The catalytic inhibition of TOP2 (Topoisomerase II) by the parent DEX^16^ as well as iron-chelation by its main metabolite (ring-opened hydrolysis product, ADR-925) that reduces the DOX-induced iron-dependent hydroxyl radical formation^24^ are the main actions of DEX attributed to its cardioprotective activity in DIC, however, the exact underlying mechanisms and whether DEX is a drug or a prodrug remain elusive and under continued investigation^25–27^. While the alteration of the DOX regimen as well as utilization of DEX have shown some clinical success in DIC, separately, a combination of the two approaches with in-depth optimization of DOX and DEX have not yet been explored completely.

The main purpose of this proof-of-concept study was to quantitatively characterize the DIC as well as the cardioprotective effects of DEX in DIC, using an in vitro model of human cardiomyocytes, by means of combining experimental data with mathematical modeling and simulation approaches. First, the study investigated experimentally the effects of DOX and DEX, as single agents and in combination, across a range of physiologically relevant concentrations in AC16 cells^28^. Subsequently, a cellular-level, mathematical toxicodynamic (TD) model was developed to capture the dynamic in vitro drug-drug interaction, and relevant parameters associated with DIC and DEX cardio-protection were estimated. Once the TD models were established, we aimed to bridge the in vitro*-*in vivo gap by linking human PK models of DOX and DEX to drive the cell-based TD models and perform simulations to evaluate the effects of long-term, clinical dosing regimens of these drugs on the relative cell viability of AC16 and determine optimal drug combinations with minimal cellular toxicity. Based on these in vitro-in vivo predictions, the Q3W (once every three weeks) DOX regimen with 10:1 DEX:DOX dose ratio over three cycles (nine weeks) was found to offer maximal cardio-protection. Overall, these simulations and the established cell-based TD models could be utilized while designing subsequent preclinical in vivo studies to better optimize DOX and DEX combinations to mitigate DIC.

## Materials and methods

### Drugs, reagents, and cell line

Doxorubicin (DOX, Adriamycin) HCl was procured from Selleck Chemicals (Houston, TX) while Dexrazoxane (DEX) was purchased from Millipore Sigma-Aldrich Co. (St. Louis, MO). DEX was dissolved in DMSO as per manufacturer’s instructions while DOX was dissolved in molecular biology grade water. All stock solutions were stored at −80 $^\circ$C and fresh serial dilutions were prepared each time prior to experiments. The current study utilized an immortalized human cardiomyocyte cell line, AC16, which was derived from primary adult human ventricular myocytes^28^. AC16 has been previously reported as potentially useful in vitro models as they retain key transcriptional factors and myogenic markers. AC16 was purchased from Millipore Sigma-EMD (St. Louis, MO) and was handled as per the manufacturer’s instructions. Cell culture reagents including Dulbecco’s Modified Eagle’s Medium (DMEM), MEM non-essential amino acids, sodium bicarbonate, Penicillin/Streptomycin, Phosphate Buffered Saline (PBS), molecular biology grade water and 0.25% trypsin/2.21 mM EDTA were purchased from Corning (Corning, NY). Dimethyl sulfoxide (DMSO), Fetal Bovine Serum (FBS) and Cell Counting kit-8 (CCK-8) were purchased from Millipore Sigma-Aldrich (St. Louis, MO).

### CCK-8 cell viability assay

AC16 cells were seeded at a density of 10X10^3^ cells per well (100 µL) of a 96-well plate and incubated for 12 h overnight to ensure adhesion. Based on preliminary concentration–response experiments as well as clinically achievable physiologically relevant concentrations^29,30^, AC16 cells were exposed to a range of concentrations of either DOX (0.5 to 10 µM), DEX (5 to 100 µM), or their combination (0.5 µM DOX with increasing concentrations 5 to 100 µM of DEX) over a time course of up to 72 h. Cell viability of AC16 was measured at 0, 12, 24, 48, 72 h for different treatment groups, using the colorimetric CCK-8 assay as per the manufacturer’s instructions. Briefly, cells were incubated in CCK-8 solution (10 µL/well of a 96-well plate) for ~ 1 h and absorbance was measured at 450 nm using a microplate spectrophotometer (Biotek, Winooski, VT). Experiments were performed in at least triplicates and compared against appropriate vehicle controls.

### Mathematical modeling

#### Development of the cellular level toxicodynamic (TD) model

**Figure 1:**
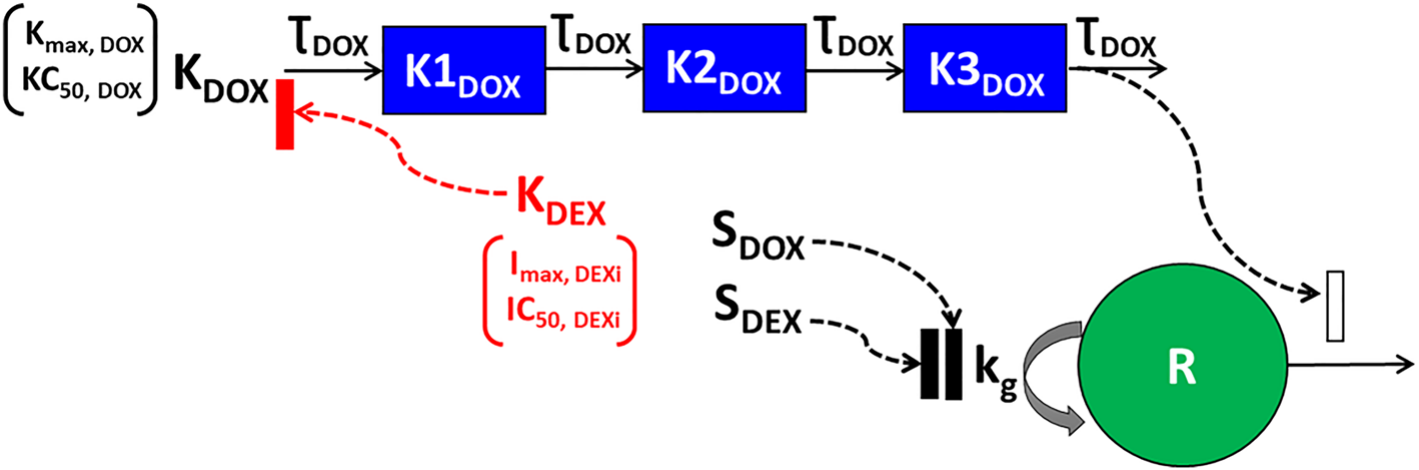
Schematic of the in vitro cellular-level toxicodynamic model (TD) for the single and combinatorial effects of Doxorubicin (DOX) and Dexrazoxane (DEX) on AC16 human cardiomyocytes. Definitions of parameters for the model are listed in Table 1. The solid lines with arrows denote turnover of the indicated response. The green circle represents cell viability while the blue boxes represent transit compartments to describe the delay in the stimulation of death by DOX. Solid black rectangles represent inhibition and open solid rectangles represent stimulation processes induced by DOX and DEX as single agents and as indicated by dashed black arrows. The cardioprotective effect of DEX on DOX-induced cardiotoxicity is indicated by red dashed arrows, with the red solid rectangle representing an inhibition process.

The schematic for the in vitro cellular-level TD model for the single agents, DOX, DEX, as well as their combinatorial effects on the cell viability of human cardiomyocytes AC16 is depicted in Fig. 1. The relevant parameters with their definitions and estimated values are listed in Table 1. The degradation profiles of DOX and DEX (Supp. Fig. 1) at 37^0^ C over a period, in cell culture media has been described previously^24,31^.

**Table 1:** Parameter estimates for the in vitro cellular-level toxicodynamic model (TD) for the single and combinatorial effects of Doxorubicin (DOX) and Dexrazoxane (DEX) on AC16 human cardiomyocytes. % RSE = % relative standard error in the model parameters.

Parameter (Units)	Definition	Estimate (% RSE)
k_g_ (hour^−1^)	First-order growth rate constant for AC16	0.0115 (2.6)
R_0_ (%)	Baseline % cell viability	101 (0.745)
S_DEX_ (µM^−1^)	Slope for the effect of DEX on the growth inhibition of AC16	0.00968 (12.1)
S_DOX_ (µM^−1^)	Slope for the effect of DOX on the growth inhibition of AC16	0.167 (13.6)
K_max, DOX_ (hour^−1^)	Maximal killing rate constant of DOX on AC16	0.0697 (1.28)
KC_50, DOX_ (µM)	DOX concentration inducing 50% of maximal killing rate on AC16	0.107 (0.858)
1/Ʈ_DOX_ (hour^−1^)	Transit constant for the stimulation of death by DOX on AC16	0.126 (2.05)
I_max, DEXi_ (hour^−1^)	Maximal inhibition rate constant for DEX on DOX’s stimulation of death	0.0625 (11.2)
IC_50, DEXi_ (µM)	DEX concentration inducing 50% of maximal inhibition rate of DEX on DOX’s stimulation of death	39 (20.6)

The equations describing the degradation kinetics of DOX and DEX are as follows:

1 $$\frac{{dC}_{DOX}}{dt}=-{k}_{deg, DOX } . {C}_{DOX},{C}_{DOX}(0)={C}_{DOX,0}$$

2 $$\frac{{dC_{DEX} }}{dt} = - k_{deg, DEX } . C_{DEX} ; C_{DEX} \left( 0 \right) \, = C_{DEX,0}$$

where, *k*_*deg,DOX*_ and *k*_*deg,DEX*_ are the first-order degradation rate constants for DOX and DEX. *C*_*DOX,0*_ and *C*_*DEX,0*_ are the initial concentrations of DOX and DEX that are used in cell viability experiments. *k*_*deg,DOX*_ and *k*_*deg,DEX*_ were assumed to be constant for all concentrations of DOX and DEX tested in the experiments. For the combination DOX + DEX group, it was assumed that the two drugs do not interfere with the degradation kinetics of each other in the cell culture media. Overall, the time dependent changes in the concentrations of DOX and DEX in the cell culture media were used to drive the cellular-level TD model described below.

Since AC16 are immortalized human cardiomyocytes, their cell growth without any treatment (control group) was best described by an exponential growth function as follows:

3 $$\frac{dR}{{dt}} = k_{g} \cdot R;R\left( 0 \right) \, = R_{0}$$

where *R* is the % cellular viability (cellular response) at time *t*, *k*_*g*_ is the first-order growth rate constant for AC16, while *R*_*0*_ is the % cellular viability at time zero.

The TD effect of DOX on the cell viability of AC16 was described by a combination of an inhibitory effect (Linear function) on the cell growth as well as a stimulatory effect (Hill function) on the cell death of AC16. The delay between the exposure of DOX and the non-linear cytotoxic (stimulation of death) effect on AC16 was captured with the inclusion of three transit compartments^32^. The effects of DOX including both inhibition of cell growth and stimulation of death as well as the temporal delay for exerting its cytotoxic effects is consistent with its mechanism of action.

The differential equations for the effect of DOX are:

4a $${K}_{DOX}=\frac{{K}_{max,DOX} . {C}_{DOX}}{{KC}_{50,DOX} +{ C}_{DOX}}$$

4b $$\frac{{dK1_{DOX} }}{dt} = \frac{1}{{\tau_{DOX} }} . \left( {K_{DOX} - K1_{DOX} } \right);K1_{DOX} \left( 0 \right) \, = \, 0$$

4c $$\frac{{dK2_{DOX} }}{dt} = \frac{1}{{\tau_{DOX} }} . \left( {K1_{DOX} - K2_{DOX} } \right);K2_{DOX} \left( 0 \right) \, = \, 0$$

4d $$\frac{{dK3_{DOX} }}{dt} = \frac{1}{{\tau_{DOX} }} . \left( {K2_{DOX} - K3_{DOX} } \right);K3_{DOX} \left( 0 \right) \, = \, 0$$

4e $$\frac{dR}{{dt}} = (1 - S_{DOX} \cdot C_{DOX} ) \cdot k_{g} \cdot R - K3_{DOX} \cdot R ;R\left( 0 \right) \, = R_{0}$$

where the inhibitory effects of DOX on the cell growth (*k*_*g*_) of AC16 is described by the linear function (1 − *S*_*DOX*_* .C*_*DOX*_). *K*_*max,DOX*_ is the maximal killing rate constant of DOX while *KC*_*50,DOX*_ is the concentration of DOX required to induce half-maximal killing of AC16 cells. *K*_*DOX*_ is the cytotoxicity function while *S*_*DOX*_ is the growth inhibition function. *K1*_*DOX*_ to *K3*_*DOX*_ are transit compartments with *Ʈ*_*DOX*_ the mean transit time between compartments.

Since a slight inhibitory effect of DEX on the cell growth of AC16 was observed, a mathematical model was developed to describe these effects. The inhibitory effect of DEX was described with a linear function (1 − *S*_*DEX*_* .C*_*DEX*_) on the cell growth (*k*_*g*_) of AC16.

The differential equations for the effects of DEX as a single agent are:

5 $$\frac{dR}{{dt}} = (1 - S_{DEX} \cdot C_{DEX} ) \cdot k_{g} \cdot R ;R\left( 0 \right) \, = R_{0}$$

Several empirical models for inhibition of cell growth due to DOX and/or DEX and stimulation of cell death due to DOX were evaluated, such as linear, power or sigmoidal Hill functions to describe the trends in the observed data. Furthermore, the number of transit compartments in the DOX model were varied for model evaluation. The final functions and transit compartments that described the observed data reasonably well were selected based on visual inspection and goodness-of-fit plots, along with good precision on parameter estimates.

#### Drug combination TD model

The TD model for the combinatorial effects of DOX and DEX is represented in Fig. 1. DEX is known to induce cardioprotective effects against DOX as also observed in drug combination studies in AC16 cells. Similar to the DOX and DEX monotherapy models, several empirical functions were evaluated in order to describe the cardioprotective effects of DEX on DOX cell-killing. In the final model, DEX cardioprotective effects were characterized by introducing an empirical, non-linear (Hill) inhibition function on DOX’ stimulation of death that theoretically captures all the underlying mechanisms of cardioprotection or opposite effects of DEX against DOX. The differential equations for the combinatorial effects of DOX and DEX in AC16 are as follows:

6a $$K_{DEXi} = \frac{{I_{max,DEXi} . C_{DEX} }}{{IC_{50,DEXi} + C_{DEX} }}$$

6b $$K_{DOX} = \frac{{K_{max,DOX} . C_{DOX} }}{{KC_{50,DOX} + C_{DOX} }}$$

6c $$\frac{{dK1_{DOX} }}{dt} = \frac{1}{{\tau_{DOX} }} \cdot \left( {(K_{DOX} - K_{DEXi} } \right) - K1_{DOX} );K1_{DOX} \left( 0 \right) \, = \, 0$$

6d $$\frac{{dK2_{DOX} }}{dt} = \frac{1}{{\tau_{DOX} }} . \left( {K1_{DOX} - K2_{DOX} } \right);K2_{DOX} \left( 0 \right) \, = \, 0$$

6e $$\frac{{dK3_{DOX} }}{dt} = \frac{1}{{\tau_{DOX} }} . \left( {K2_{DOX} - K3_{DOX} } \right);K3_{DOX} \left( 0 \right) \, = \, 0$$

6f $$\frac{dR}{{dt}} = (1 - (S_{DOX} \cdot C_{DOX} + S_{DEX} \cdot C_{DEX} )) \cdot k_{g} \cdot R - K3_{DOX} \cdot R ;R\left( 0 \right) \, = R_{0}$$

where *I*_*max,DEXi*_ is the DEX’s maximal inhibition on DOX’s stimulation of death while *IC*_*50,DEXi*_ is the concentration of DEX required to induce 50% of *I*_*max,DEXi*_ in AC16 cells.

### In vitro*–*in vivo translation of toxicodynamic response

In vitro to in vivo translation of TD response for the joint effect of DOX + DEX in cardiomyocytes was performed by utilizing clinically relevant pharmacokinetic (PK) profiles of the two agents to drive TD models developed in the in vitro setting. The PK data of DOX were described using a three-compartment model as described by Kontny N.E. and co-workers^30^ at a clinically relevant DOX dose of 50 mg/m^2^ administered once every three weeks (Q3W) as a 15-min intravenous infusion.

The resulting typical values of DOX PK model parameters (Table 1) for a subject having a body surface area of 1.8 m^2^ were used for model simulations. For PK model development of DEX, serum concentration data under various intravenous infusion regimens were extracted from Earhart R.H. and co-workers^29^. A two-compartment model was used to adequately fit the data, which was further used to simulate the PK of DEX at doses ranging from 50–2500 mg/m^2^, as a Q3W 15-min intravenous infusion. The model equations governing the PK model for DEX are as follows:

$$\frac{dA1}{dt}=DE{X}_{Infusion}-kel.A1-k12.A1+k21.A2 ;A1\left(0\right)=0$$

$$\frac{dA2}{dt}=k12.A1-k21.A2 ;A2\left(0\right)=0$$

$$C1=A1/V$$

where *DEX*_*Infusion*_ represents the short-term infusion rate of DEX, *A1* represents amount of drug in the central compartment, *A2* represents amount of drug in the peripheral compartment, *kel* is the first-order elimination rate constant from the central compartment, *k12* and *k21* are the inter-compartmental distribution rate constants, *V1* is the volume of the central compartment and *C1* is the concentration in the central compartment. The simulated in vivo PK profiles for DOX and DEX were used to drive the developed in vitro TD model for DOX + DEX to predict the in vivo TD counterpart. Model-based simulations using a nonlinear mixed effect modeling approach were conducted for 500 subjects by introducing an arbitrary inter-individual variability (IIV) of 10% on the TD parameters. The evaluation of toxicity was performed by calculating the area under the effect (cell viability) curve (AUEC) as an integrated measure of TD response for various dose ratios of DEX:DOX ranging from 0:1 to 50:1, in order to determine the optimal dose ratio eliciting maximum cardioprotection. Furthermore, TD simulations were performed by fractionating the 50 mg/m^2^ Q3W DOX dose into a 16.67 mg/m^2^ once every week (Q1W) dose, in order to evaluate a combination of DOX dose-fractionation and DEX treatment as a strategy to enhance cardioprotection.

All PK-TD model fittings and simulations were performed using Monolix suites version 2016R1 or higher, while AUEC calculations were performed using RStudio version 1.2.5033.

## Results

### Single and combinatorial effects of DOX and DEX on human cardiomyocytes

**Figure 2:**
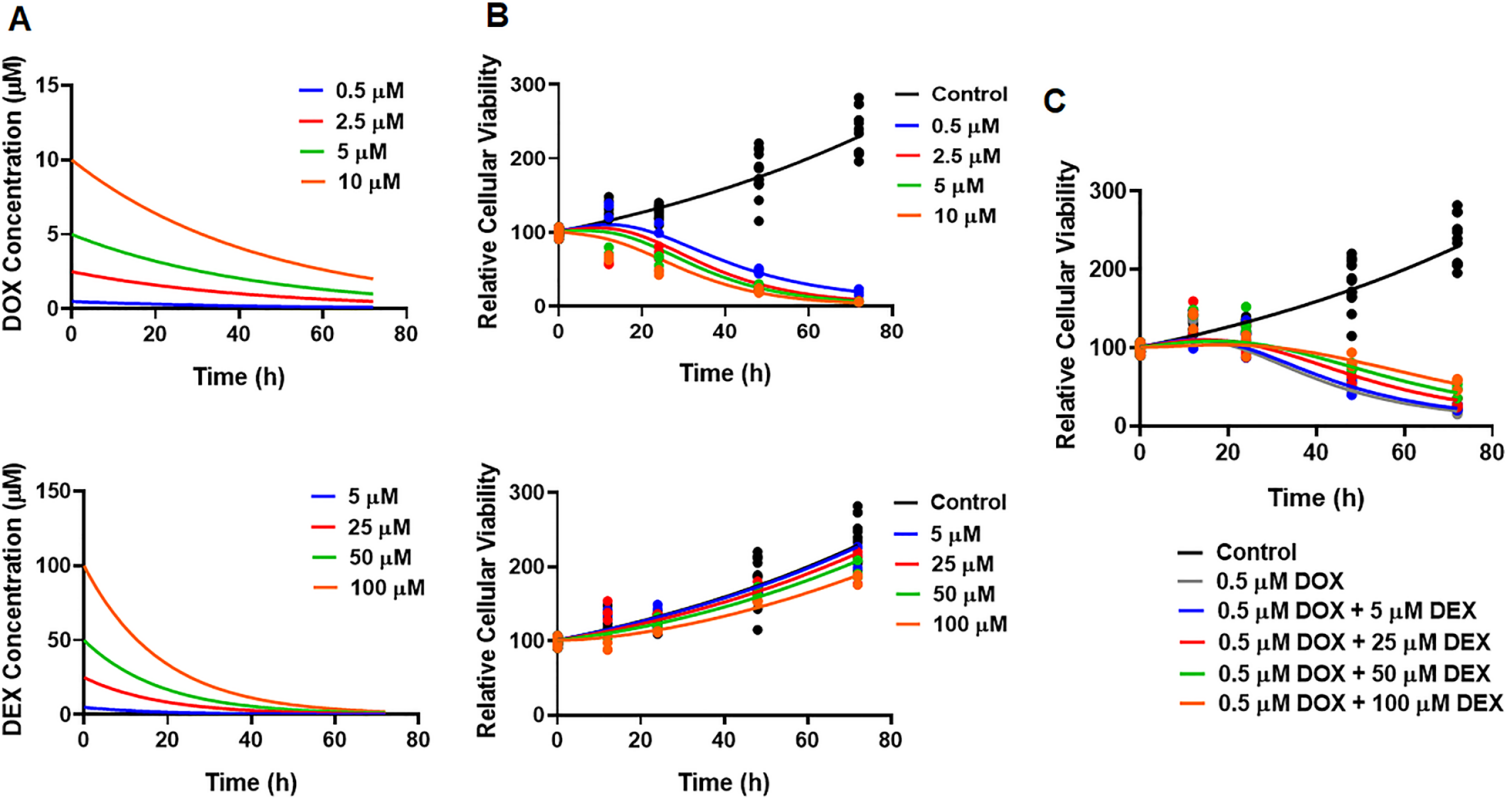
(**A**) Simulated degradation kinetics of DOX (*top*) and DEX (*bottom*) at indicated concentrations in a cell culture media. The first-order degradation rate (k_deg_) for DOX and DEX were estimated as in Supp. Fig. 1 and were assumed to be constant at indicated concentrations. (**B**, **C**). Model fittings for the in vitro effects of the single agents, DOX (**B**, top) and DEX (**B**, *bottom*) as well as the combinatorial effects of DOX and DEX (**C**) at indicated concentrations over time on the cell viability of human cardiomyocytes AC16s (toxicodynamic model). All observed data are represented by solid circles while the smooth lines are model fittings or simulations.

The AC16 cell line was exposed to a range of concentrations for single agents DOX, DEX, and the combination, which were physiologically relevant (i.e., achievable in humans) as well as based on a pilot in vitro study (*data not shown*). A mathematical TD model (Fig. 1) was developed to describe the time-course effects of drugs, single agents and combination, on the cell viability of AC16. As previously noted, there is a loss of both, DOX and DEX in cell culture media over time^24,31^. The first-order degradation rate constants for DOX (k_deg, DOX_) and DEX (k_deg, DEX_) were used to describe the decline of the respective drug concentrations over time via degradation models (Eqs. 1 and 2). Based on previous studies, k_deg, DOX_ and k_deg, DEX_ were estimated to be 0.022 (± 0.0004) h^−1^ and 0.054 (± 0.0016) h^−1^, respectively (Supp. Fig. 1). These degradation rate constants were then used to simulate the expected concentration profiles of DOX and DEX over time for different concentrations used in the study (Fig. 2A). The simulated drug concentration profiles were subsequently used to drive the cellular-level TD model (Fig. 1) to account for loss of drug over time.

In the absence of the drugs, the cell viability of AC16 over time was characterized with an exponential growth function (Eq. 3) with a first-order growth rate constant (kg) which was estimated at 0.0115 (± 0.0002) h^−1^. For single agent DOX, a combination of inhibitory effects on cell growth and stimulatory effects on cell death adequately characterized the dynamic changes of AC16 cell viability over 72 h (Eq. 4a–e). While the inhibitory effect on cell growth was described with a linear function, S_DOX_ on kg (Eq. 4e), the stimulatory effect on cell death was characterized with a capacity-limited, Hill function, K_DOX_ (Eq. 4a–e). The estimated, maximal killing rate constant (K_max, DOX_) for DOX in AC16 was 0.0697 (± 0.0008) h^−1^ while the DOX concentration inducing 50% of maximal cell killing (KC_50, DOX_) was estimated to be 0.107 (± 0.0009) µM. The delayed effect of DOX was captured well with the help of three transit compartments on the stimulation of death function, with Ʈ_DOX_ representing the mean transit time for each compartment, which was estimated to be ~ 8 h. In addition, the inhibitory effect on AC16 cell growth by single agent DEX, was adequately described by a linear function, S_DEX_ on kg (Eq. 5). To characterize the time-course combinatorial effects of DOX + DEX on the cell viability of AC16, the model structure incorporated the inhibitory effect of DEX on DOX stimulation of cell death along with the inclusion of the individual model structure components. Here, the inhibitory effect of DEX was characterized via a capacity limited Hill function, K_DEX_ (Eq. 6a-6f), as depicted by the red solid rectangle in Fig. 1. The IC50 for DEX inhibitory effect on DOX cell-death stimulation was estimated at 39 (± 8.034) µM and its maximal fraction of inhibition was estimated at 0.0625 (± 0.007).

Overall, the models characterized well the observed delayed and dose-dependent killing of DOX single agent (Fig. 2B), slight growth inhibition of DEX single agent (Fig. 2B), as well as significant protective effects of DEX in a dose-dependent manner (Fig. 2C) (at a particular concentration of DOX with increasing concentrations of DEX). Simultaneous fitting of all data (single agents and combination) with Eqs. (3–6) resulted in estimation of all parameters with good precision (Table 1, Supp. Fig. 2). The parameter estimates for single agents as well as combination are summarized in Table 1.

### Optimization of DOX and DEX combinations with simulations for clinically relevant dosing regimens

**Figure 3:**
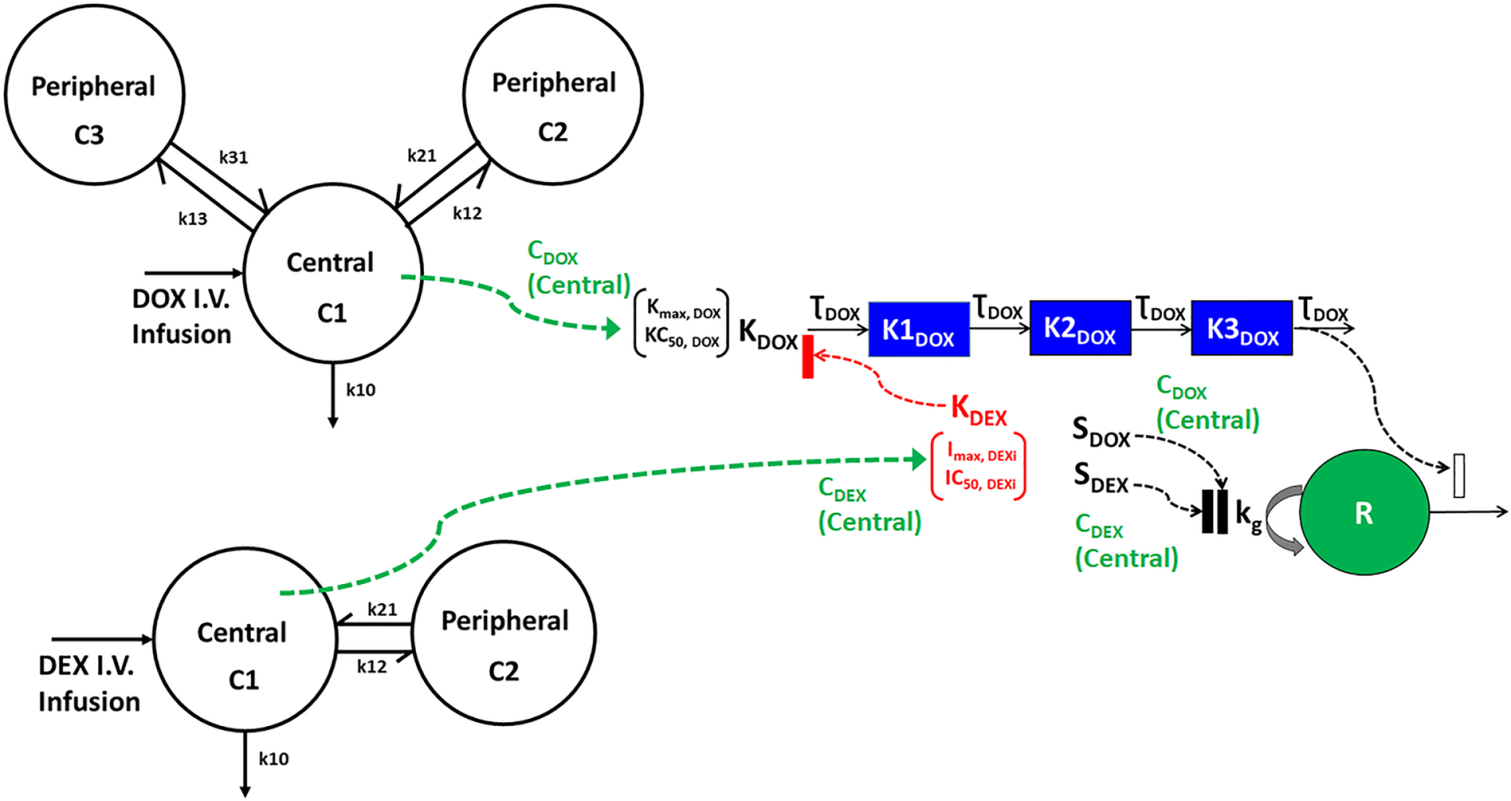
Model structure of clinical pharmacokinetics for doxorubicin (DOX) (3-compartment; *Left, Top*) and for Dexrazoxane (DEX) (2-compartment; *Left, Bottom*). Schematic of the in vitro cellular-level toxicodynamic model (TD) for the single and combinatorial effects of Doxorubicin (DOX) and Dexrazoxane (DEX) (*Right*) on human cardiomyocytes. Time-varying concentrations were utilized to drive the DOX + DEX TD model as represented by the green dashed arrows.

The cellular-based TD models for DOX and DEX treatments in AC16 cells described well all the data via simultaneous fitting of the single agents and combinations with a reasonable estimation of the parameters. As a next step, the TD models were used to perform simulations and investigate the effects of long-term, clinical dosing regimens on the cell viability of AC16. To this end, the clinical PK models of DOX and DEX were used to predict the change in plasma drug concentrations over time and drive the cellular TD models (Fig. 3) to optimize the drug combination with minimal cellular toxicity (i.e., DIC). PK of DOX was characterized with a previously published three-compartment mammillary model (Fig. 3) with linear elimination^30^. On the other hand, for DEX, a two-compartment mammillary model (Fig. 3, Supp. Fig. 3) with linear elimination, was used to describe the observed PK data from a Phase I trial^29^. All parameter estimates for DOX and DEX PK are summarized in Table 2.

**Table 2:** Parameter estimates for the clinical pharmacokinetic (PK) models for DOX (*top*) and DEX (*bottom*). % RSE = % relative standard error in the model parameters.

Parameter (Units)	Definition	Estimate (% RSE)
Clinical pharmacokinetic model parameters for doxorubicin		
CL (L/h/1.8m^2^)	Typical value of clearance from the central compartment	53.3 (4)
V (L/1.8m^2^)	Typical value of volume of central compartment	17.7 (8)
Q2 (L/h/1.8m^2^)	Typical value of inter-compartmental clearance between central and peripheral compartment 1	58.7 (8)
V2 (L/1.8m^2^)	Typical value of volume of peripheral compartment 1	1830 (7)
Q3 (L/h/1.8m^2^)	Typical value of inter-compartmental clearance between central and peripheral compartment 2	21.8 (13)
V3 (L/1.8m^2^)	Typical value of volume of peripheral compartment 2	71.6 (15)
Clinical pharmacokinetic model parameters for dexrazoxane		
kel (h^−1^)	Elimination rate-constant for dexrazoxane elimination from the central compartment	1 (4)
k12 (h^−1^)	First-order rate constant for distribution of drug from the central to the peripheral compartment	1 (3)
k21 (h^−1^)	First-order rate constant for distribution of drug from the peripheral to the central compartment	1 (8)
V (L)	Volume of the central compartment	14.6 (5)

**Figure 4:**
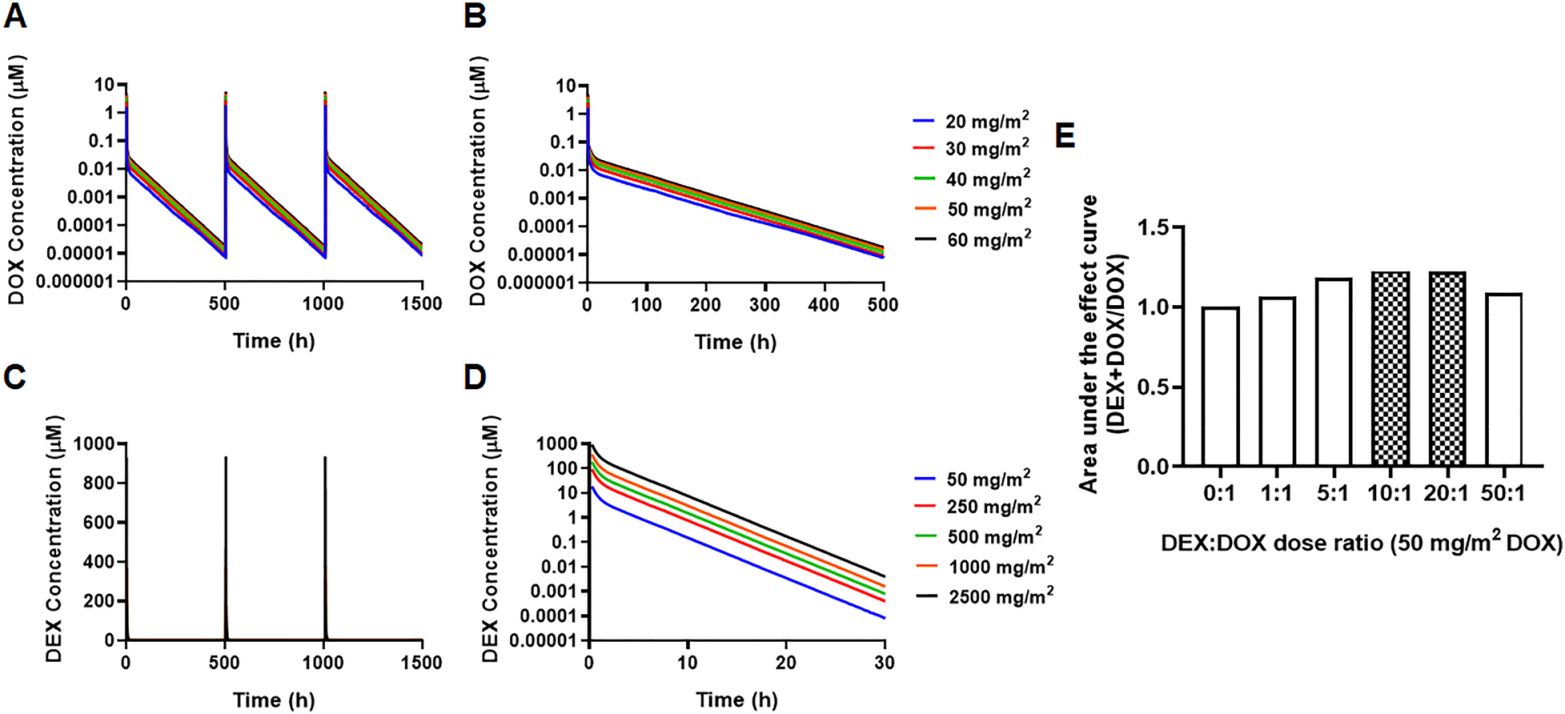
(**A**,**B**) Model simulations for doxorubicin (DOX) clinical pharmacokinetics over three dosing cycles (**A**) and one dosing cycle (**B**) at indicated dose levels. (**C**,**D**) Model simulations for dexrazoxane (DEX) clinical pharmacokinetics over three dosing cycles (**C**) and one dosing cycle (**D**) at indicated dose levels. (**E**) Area under the effect (cell viability) curves for 50 mg/m^2^ DOX administered once every three weeks for three dosing cycles in the presence of varying DEX:DOX dose ratios as indicated. AUECs are represented as the ratio of AUEC_DEX+DOX_/AUEC_DOX._. *The shaded bar represents the optimal dose ratio that demonstrates maximum cardio protection with DEX.*

**Figure 5:**
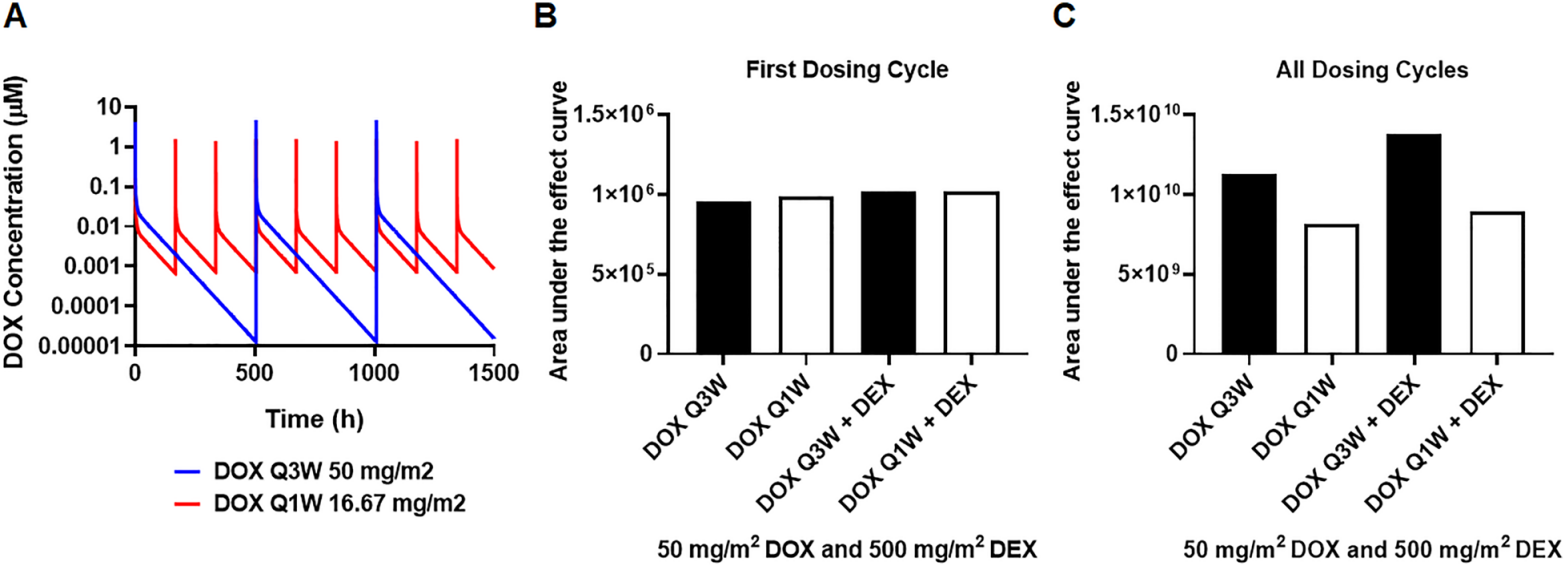
(**A**) Doxorubicin (DOX) clinical pharmacokinetic simulations: Blue profile—50 mg/m^2^ administered every three weeks (Q3W) for three cycles, Red profile—16.67 mg/m^2^ administered once every week (Q1W) as a dose-fractionated regimen. (**B**) Area under the effect (cell death) curves for doxorubicin (DOX) administered as a Q3W or Q1W regimen in the presence or absence of dexrazoxane (DEX) over three weeks (one dosing cycle) and (**C**) over nine weeks (three dosing cycles). Black solid bars represent Q3W regimens and open bars represent Q1W regimens of DOX. *The Q3W DOX regimen with 10:1 DEX:DOX ratio at the indicated dose levels demonstrates maximum cardio protection.*

Using the established PK models, the drug PK profiles were simulated for various clinically relevant DOX and DEX dosing regimens, wherein both drugs were administered simultaneously every three-weeks (Q3W) for three cycles over 15-min infusions. These simulations included varying doses of DOX (20, 30, 40, 50, 60 mg/m^2^) as shown in Fig. 4A-B, and the corresponding doses for DEX at DEX:DOX dose ratios of 1:1, 5:1, 10:1, 20:1 and 50:1 (representative DEX profiles shown in Fig. 4C,D). To determine optimal drug combination ratios with maximal cardioprotection, the simulated PK profiles were used to drive the cellular TD model (Fig. 3) and the area under the effect (cell viability) curves (AUEC) was calculated for DOX single agent (AUEC_DOX_), and in combination with DEX (AUEC_(DOX+DEX)_) treatment. For each DEX:DOX dose ratio, the ratio of AUEC_(DOX+DEX)_/ AUEC_DOX_ was calculated to quantitate the protective effects of DEX on DOX treatment. The simulation predicted maximal protective effects of DEX at higher doses of DOX (50, 60 mg/m^2^) as well as at later dosing cycles (cycle 3) (*data not shown*). As shown in Fig. 4E, the DEX:DOX dose of 10:1 or 20:1 was predicted to provide maximal cardioprotective effects. Finally, PK simulations were carried out for DOX dose-fractionation, wherein the 50 mg/m^2^ DOX dose administered every three weeks (Q3W), was fractionated into 16.67 mg/m^2^ given once every week (Q1W) (Fig. 5A), in absence or presence of 500 mg/m^2^ DEX (optimal DEX:DOX ratio of 10:1) and AUEC was calculated as above. As shown in Fig. 5B,C, the Q3W DOX regimen with 10:1 DEX:DOX dose ratio over three cycles (nine weeks) was found to offer maximal cardio-protection. No significant improvement in cell viability was observed with DOX dose-fractionation alone or with the drug combination at the end of the first cycle (three weeks).

## Discussion

In-spite of the high anticancer activity, dose-limiting cardiotoxicity hinders the extensive utility of DOX in a clinical setting^4,5^. Since various mechanisms have been postulated, various strategies have also been investigated to overcome DIC^7^. However, DEX remains the only cardioprotective agent to be approved for the treatment of anthracycline-induced cardiomyopathies^22,23^. Besides DEX, modulating the dosing regimen of DOX (eg. Dose fractionation) itself has proved to be beneficial in decreasing the risk of DIC^20,21^. However, both approaches have limitations and further studies are required to better optimize the two approaches in combination for maximal beneficial effects. Hence, in this present work, we first aimed to quantitatively capture DIC as well as the protective effects of DEX with a mathematical, cell-based TD model. Subsequently, we executed in vitro*–*in vivo translation by simulating clinical PK profiles for different dosing regimens of DOX, alone and in various combinations with DEX, and used the simulated PK profiles of drugs to drive the cell-based TD models. Finally, we identified that the Q3W (once every three weeks) DOX regimen with 10:1 DEX:DOX dose ratio over three cycles (nine weeks) may offer maximal cardio-protection. Overall, the cell-based TD model can be effectively used to simulate different scenarios and to better design subsequent preclinical studies aimed for further optimizing safe and effective DOX and DEX combinations.

We first developed a cell-level TD model by evaluating the time-course effects of single agents, DOX and DEX, as well as their combinations, on the cell viability of AC16. Previous studies have reported degradation of both, DOX and DEX, in cell culture media^24,31^. Hence, to account for the change in concentrations of these drugs over time, we incorporated respective, in vitro degradation models to drive TD responses. Here, we assumed that the respective degradation rate constants are consistent across all concentration levels of DOX and DEX, and that the two drugs do not impact each other’s degradation profiles when used as combination in the cell culture media. While the physical drug-drug interaction of DOX with DEX in the cell culture media was not investigated in the present study, the above assumption is based on previous studies that reported no impact of DEX on the PK of DOX in a preclinical rat model as well as in breast cancer patients^33^.

Next, the cardiotoxic effects of DOX and the cardioprotective effects of DEX were quantitatively characterized, across a range of physiologically relevant concentrations. Accordingly, we used a lower µM (0.5 to 10) concentration-range for DOX in the in vitro studies. Besides, we also observed that the cytotoxic effects of DOX in AC16 was significantly delayed below 0.5 µM concentration. In such a scenario, simultaneous co-treatment with DEX (5 to 100 µM) did not offer any cardioprotective effects in AC16 (*data not shown*), possibly due to a much faster degradation rate in cell culture media compared with DOX (k_deg, DOX_ and k_deg, DEX_ estimated to be 0.022 (± 0.0004) h^−1^ and 0.054 (± 0.0016) h^−1^). Hence, a lower µM (0.5 to 10) concentration-range was used to also ensure a rapid onset of action for DOX in AC16. Similarly, physiologically-relevant, higher µM (5 to 100) concentration-range for DEX was included to test its cardioprotective effects at various DEX:DOX ratios (10:1 to 200:1) and at a fixed, more representative physiological concentration of DOX (0.5 µM).

Although DEX exhibited dose-dependent cardio-protective effects, it was not able to completely reverse or suppress the cardiotoxicity of DOX in AC16 (Fig. 2C). In addition, DEX itself induced minimal growth inhibition in AC16, especially at higher concentrations (Fig. 2B), which was also observed in previous studies^26^. This minimal growth inhibition of DEX in AC16 was also accounted for within the developed TD model. Previous studies suggested a correlation for loss of AC16 cell viability and release of cardiotoxicity biomarkers such as BNP (Brain Natriuretic Peptide) in the in vitro setting^31^. Hence, the loss of cell viability was considered representative of DOX-induced cardiotoxicity in the current study. Regardless, the use of relevant biomarkers of cardiotoxicity, including BNP and Troponin^34,35^, as well as exploring other cell lines such as iPSC-derived cardiomyocytes (as against immortalized cardiomyocytes, AC16) could be some of the considerations for future studies^36^. Taken together, these results reiterate the need for further in-depth evaluation of DEX as a single agent and an optimized combination with DOX to overcome DIC, in a preclinical setting.

Finally, we extended the cell-based TD models to perform clinical simulations and predict the effects of long-term dosing regimens on the cell viabilty of AC16. Overall, the main goal here was not to predict the clinical response, but to utilize this strategy of in vitro*-*in vivo translation to identify optimized DEX:DOX ratios as well as dosing regimens for the combination that can offer maximal cardio-protection, which can then be tested preclinically. This can substantially help in better designing of future preclinical studies and better selection of DOX and DEX treatment groups. Consistently, we predicted the DEX:DOX dose of 10:1 or 20:1 to provide maximal cardioprotective effects. In addition, DOX Q3W (total dose administered every three weeks) was predicted to offer more cardioprotective effects as compared with DOX Q1W (fractionated dose administered every week), in the presence or absence of DEX. Efforts are ongoing to leverage previously developed more mechanistic, PBPK (Physiologically-Based Pharmacokinetic) models^31,37^ to characterize tissue (e.g. heart) concentrations (over time) for DOX and DEX to better predict TD and DEX’s cardioprotective effects^31^ in a preclinical and clinical setting.

## Conclusion

To summarize, we have developed a proof-of-concept cell-based TD model which can serve as a platform to optimize DOX and DEX combinations to counteract DIC. Efforts are ongoing to extend the PK model to include different tissue compartments including the heart and link the cardiac drug concentrations for the TD predictions. Similarly, capturing the dynamic changes in the intracellular signaling pathway for DOX and DEX TD models can enable better quantitative understanding of the underlying mechanisms involved in DIC and DEX-induced cardioprotection. Such developed PK/PD models can serve as in silico tools to assess DIC and design better preclinical in vivo studies for DOX and DEX combinations.

## Supplementary Information

Supplementary Information.

## Data Availability

The datasets used and/or analysed during the current study available from the corresponding author on reasonable request.
