# Supplementary material for: In vitro to clinical translational pharmacokinetic/pharmacodynamic modeling of doxorubicin (DOX) and dexrazoxane (DEX) interactions: Safety assessment and optimization
Source: Sci Rep. 2023 Feb 22;13:3100. doi: 10.1038/s41598-023-29964-4 (PMC9947016; doi:10.1038/s41598-023-29964-4)
Supplement: Supplementary file 1 — Supplementary Information. [file 41598_2023_29964_MOESM1_ESM.docx]

**Supplementary Material**

***In Vitro* to Clinical Translational Pharmacokinetic/Pharmacodynamic Modeling of Doxorubicin (DOX) and Dexrazoxane (DEX) Interactions - Part I: Safety Assessment and Optimization**

Hardik Mody^1#^, Tanaya R. Vaidya^1#^, and Sihem Ait-Oudhia^2^

# Contributed equally

^1^Center for Pharmacometrics and Systems Pharmacology, Department of Pharmaceutics, College of Pharmacy, University of Florida, Florida, USA.

^2^ Quantitative Pharmacology and Pharmacometrics (QP2), Merck & Co., Inc, Kenilworth, New Jersey, USA.

**Corresponding author:** Sihem Ait-Oudhia, MS., PharmD., PhD. Quantitative Pharmacology and Pharmacometrics (QP2), Merck & Co., Inc, Kenilworth, New Jersey, USA. Address: 2000 Galloping Hill Rd, Kenilworth, NJ 07033. Email: sb.manuscript.submission@gmail.com

**
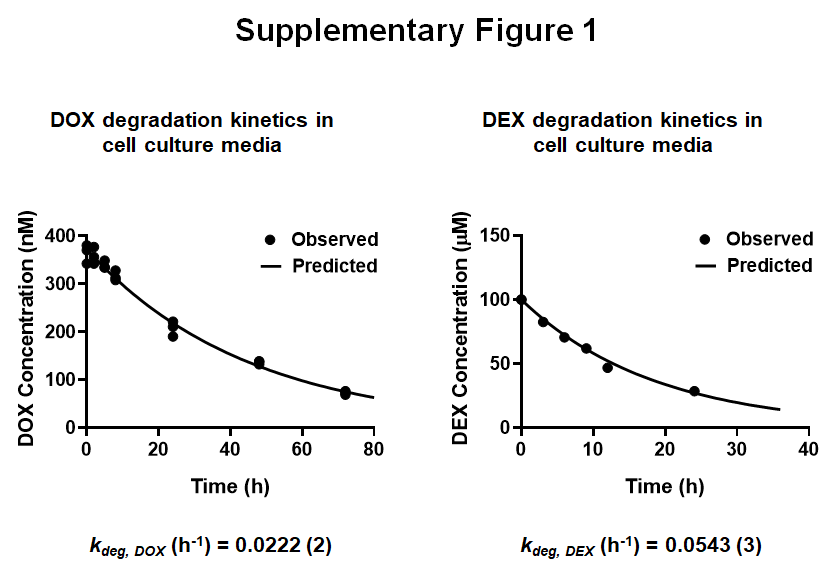
**

**
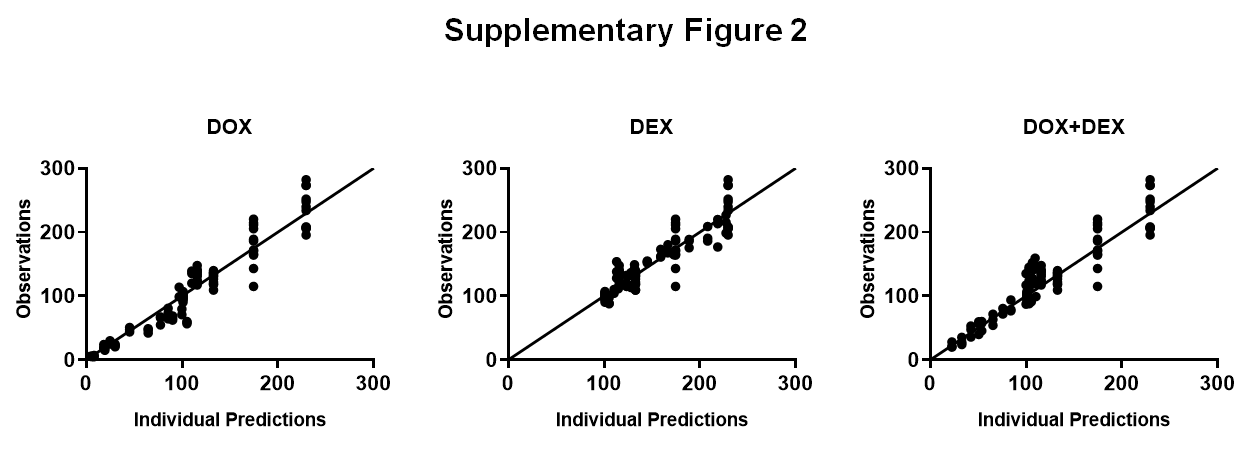
Supplementary Figure 1.** The degradation kinetics of DOX (*left*) and DEX (*right*) in cell culture media based on the data reported previously (ref). The estimated first-order degradation rate constants for DOX (K_deg, DOX_)and DEX (K_deg, DEX_) were assumed to be constant for all concentrations for the two drugs.

**Supplementary Figure 2.** Observations vs. individual prediction plots for the cell viability (toxicodynamic) fittings for DOX (*left*), DEX (*middle*), and DOX+DEX (*right*). The black solid circles represent observed data while the solid line represent identity line with y=x.

**
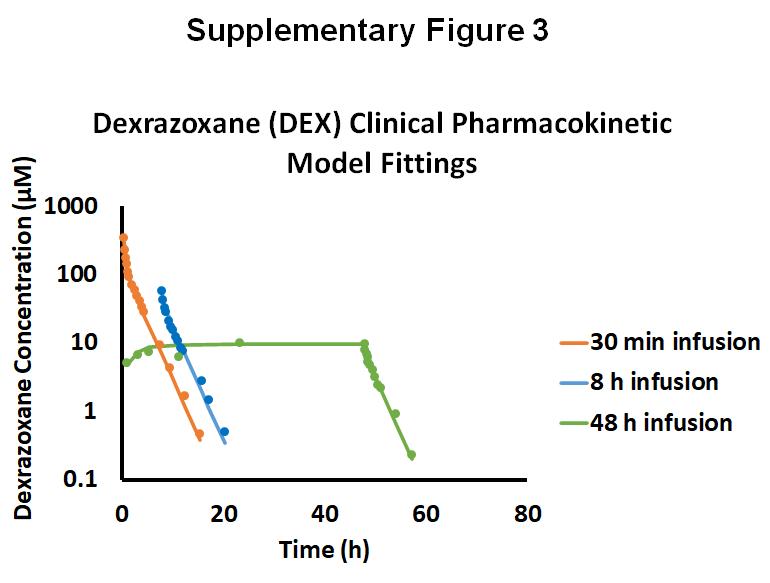
**

**Supplementary Figure 3.** Model fittings for clinical pharmacokinetics of dexrazoxane (DEX) based on the data collected from *Earhart RH, Tutsch KD, Koeller JM et al. Cancer Res. 1982 42(12):5255-61*. Solid circles represent observed data and smooth curves represent fitted profiles for various infusions of 1000 mg/m^2^ dexrazoxane (DEX).
